# Current Applications of Metabolomics in Cirrhosis

**DOI:** 10.3390/metabo8040067

**Published:** 2018-10-22

**Authors:** Vinshi Khan, Nagireddy Putluri, Arun Sreekumar, Ayse L. Mindikoglu

**Affiliations:** 1 Margaret M. and Albert B. Alkek Department of Medicine, Section of Gastroenterology and Hepatology, Baylor College of Medicine, Houston, TX 77030, USA; Vinshi.Khan@bcm.edu; 2 Department of Molecular and Cellular Biology, Baylor College of Medicine, Houston, TX 77030, USA; putluri@bcm.edu (N.P.); Arun.Sreekumar@bcm.edu (A.S.); 3 Michael E. DeBakey Department of Surgery, Division of Abdominal Transplantation, Baylor College of Medicine, Houston, TX 77030, USA

**Keywords:** cirrhosis, metabolomics, liver, mortality

## Abstract

Metabolomics is the identification and quantification of all or specified metabolites in a living system under a specific condition or disease. Metabolomics in cirrhosis can be used in diagnosing complications, determining prognosis and assessment of response to therapy. In this review, we summarized representative applications of metabolomics in cirrhosis and significant metabolites associated with cirrhosis and its complications.

## 1. Introduction

### 1.1. Metabolomics, Metabolome, Metabolite

**Figure 1:**
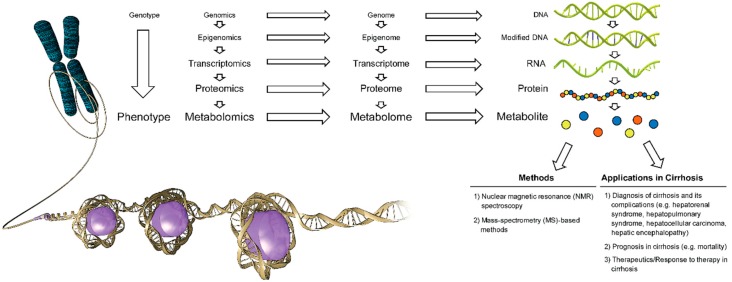
Current methods and applications of metabolomics in cirrhosis (*Used with permission of Baylor College of Medicine*).

Metabolites are endogenous or exogenous small low-molecular weight downstream intermediate or end-products of genes and proteins in a living organism. The composition of all metabolites generated by a system in a living organism (e.g., cell, organ, tissue) constitute a metabolome [1,2]. Metabolomics is the identification and quantification of all (non-targeted metabolomics profiling) or specified metabolites (targeted metabolomics profiling) in a biological sample (e.g., blood, urine) under a specified condition or disease and identification of metabolic pathways and genes associated with the measured metabolites [1,2] (Figure 1).

### 1.2. Major Analytical Techniques in Metabolite Detection, Quantitation and Data Analysis

Identification of metabolites in biological samples requires two major analytical consecutive steps including separation of metabolites using gas chromatography, high-performance liquid chromatography or capillary electrophoresis followed by identification and quantification of metabolites using mass spectrometry (MS) or nuclear magnetic resonance (NMR) spectroscopy [1,2,3,4]. Both techniques have strengths and limitations in metabolomic profiling (Table 1) [1,2,3,4]. MS is more sensitive and specific for metabolite detection and more affordable compared to NMR spectroscopy, however it requires separation of analytes for detection and identification [1,2,3,4]. In contrast, NMR spectroscopy is less sensitive for metabolite detection, generally limited to less than 100 analytes in biological fluids, less affordable compared to MS, however it does not require separation of analytes by chromatography or capillary electrophoresis for detection [1,2,3,4].

Detection and quantitation of the analytes using MS is dependent on separation of analytes prior to MS analysis [1,2,3,4]. There are different separation techniques based on metabolite that can be coupled with MS [1,2,3,4]. Liquid chromatography (LC), gas chromatography (GC) and capillary electrophoresis (CE) are among the most widely used techniques [1,2,3,4]. Prior to MS analysis, metabolic extraction using sequential application of ice-cold organic and aqueous solvents, deproteinization and drying of the extract are performed [1,2,3,4]. Metabolite separation using liquid chromatography requires application of different mobile phases specific to different metabolites. Therefore, samples from the same experiments (e.g., plasma sample collected from a study participant) are aliquoted into multiple fractions to be analyzed in different chromatographic conditions [1,2,3,4]. These different chromatographic methods are applied to measure various classes of metabolites using MS (platform) [1,2,3,4].

After chromatographic separation, the MS step starts with ionization of the analytes [1,2,3,4,5]. Several ionization techniques have been described based on the chromatographic method used (e.g., electron or chemical ionization used in gas chromatography; electrospray ionization (ESI), atmospheric pressure chemical ionization used in liquid chromatography) [1,2,3,4]. ESI is among the most commonly used technique in metabolomics [1,2,3,4].

For data analysis, the platform displaying the best characteristics is chosen. Raw data consist of integrated peak areas quantified by the area under the receiver operating characteristic curve, where the units are ion counts [1,2,3,4]. Metabolites are identified based on their retention time on liquid chromatography and mass-to-charge ratio (*m*/*z*) obtained on MS [1,2,3,4]. Prior to statistical analysis, different normalization and transformation techniques are applied to transform data into normal scaled data [1,2,3,4]. Metabolite datasets may contain missing values due to low metabolite concentrations requiring imputation with the lowest detected metabolite value in the sample cohort [1,2,3,4].

After the metabolomic data are pre-treated (i.e., normalization, transformation), univariate and multivariate analysis can be performed by different techniques. The primary goal of metabolomics data analysis is metabolite biomarker discovery for disease diagnosis and treatment. Univariate analysis techniques such as *t*-tests, analysis of variance (ANOVA), volcano plots (plots log of fold change in metabolite(s) value(s) versus negative log of *p* value) examine a single variable of interest (metabolite) [5]. In contrast to univariate analysis, multivariate analysis examines relationships among multiple variables of interest (several metabolites). Commonly applied multivariate analysis techniques include principle component analysis (PCA), partial least-squares regression (PLS) and discriminant analysis (PLS-DA), and orthogonal partial least-squares regression (OPLS) and discriminant analysis (OPLS-DA) [5]. PCA is a data reduction technique that recognizes meaningful metabolite patterns, and plots them in a 2-D graph [5]. PLS and OPLS are regression methods to develop a predictive model [5,6]. Metrics used for interpretation of a model include *R*^2^*X*, *R*^2^*Y*, and *Q*^2^*Y* that range between 0 and 1 [5,6]. *R*^2^*X* and *R*^2^*Y* are percentages of the variance in the independent and dependent variables, respectively explained by the model [5,6]. *Q*^2^*Y* is the estimated predictive accuracy of the model [5,6]. High accuracy models would have values of *R*^2^*X*, *R*^2^*Y*, and *Q*^2^*Y* close to 1 [5,6].

In addition to univariate and multivariate analysis techniques, pathway and network analysis is performed to identify alterations in metabolic pathways and gain a more profound insight in complex disease processes [7,8].

### 1.3. Metabolomics in Cirrhosis

Several metabolomic studies were conducted in patients with cirrhosis to identify metabolomic signatures related to the cause and complications of cirrhosis including decompensation, acute-on-chronic liver failure, hepatorenal dysfunction, hepatic encephalopathy, hepatocellular carcinoma, prognosis and response to treatment (Table 2). Herein, we summarized methods and findings of several metabolomic studies conducted in patients with cirrhosis.

## 2. Current Applications of Metabolomics in Cirrhosis

### 2.1. Differentiation between Patients with Cirrhosis and Healthy Controls

Studies showed that a set of metabolites is capable of differentiating patients with cirrhosis from healthy controls. Qi et al. [9] conducted a metabolomic pilot study among 60 hepatitis B surface antigen positive patients with compensated and decompensated cirrhosis, and 30 healthy controls using ^1^H NMR-spectroscopy and performed PCA and OPLS-DA for data analysis. Their results showed that there was a distinct metabolomic signature that differentiated patients with cirrhosis from healthy controls (*R*^2^*Y* = 0.941, *Q*^2^*Y* = 0.836) [9]. This metabolomic signature included several metabolites (e.g., alpha- and beta-glucose, glutamine, glutamate, isobutyrate, lipid, low density lipoprotein (LDL), succinate, tyrosine, valine, very low-density lipoprotein (VLDL)) [9].

In another study conducted among 41 patients with hepatitis B and alcoholic cirrhosis and 20 healthy controls, Qi et al. [10] reported that similar metabolomic signatures detected using ^1^H NMR-spectroscopy differentiated patients with cirrhosis with hepatitis B (*R*^2^*Y* = 0.915, *Q*^2^*Y* = 0.887) and alcoholic cirrhosis (*R*^2^*Y* = 0.926, *Q*^2^*Y* = 0.903) from healthy controls.

Similarly, Gao et al. [11] using ^1^H NMR-spectroscopy reported significantly decreased acetoacetate, choline, isoleucine, LDL, leucine, unsaturated lipid, valine, and VLDL, and increased acetate, α-ketoglutarate, glycerol, glutamine, 1-methylhistidine, *N*-acetylglycoproteins, phenylalanine, pyruvate, taurine, and tyrosine, in 36 patients with cirrhosis compared to 63 healthy controls (*R*^2^*Y* = 0.512, *Q*^2^*Y* = 0.881). 

Using quantitative hepatic phosphorus-31 magnetic resonance spectroscopy, Corbin et al. [12] reported significantly lower hepatic beta-ATP and higher phosphomonoester/phosphodiester ratio in 6 patients with decompensated cirrhosis compared with 8 healthy controls. Results of this study suggested reduction in energy stores and pathological changes in phospholipid metabolism in subjects with decompensated cirrhosis [12].

Taken altogether, although small sample size was the major limitation of these studies, their findings suggest presence of altered lipid metabolism, tricarboxylic acid cycle, and energy metabolism in patients with cirrhosis compared to healthy individuals [9,10,11,12].

### 2.2. Differentiation between Decompensated Cirrhosis and Compensated Cirrhosis

Qi et al. [9] showed that there was a distinct serum metabolomic signature that differentiated decompensated from compensated cirrhosis (*R*^2^*Y* = 0.784, *Q*^2^*Y* = 0.598, validation with an accuracy of 85%) [9]. Significant metabolome differences were obtained when patients with compensated cirrhosis were compared to those with decompensated cirrhosis; the latter showed higher levels of alanine, creatine, glutamate, glutamine, histidine, lysine, phenylalanine, pyruvate, succinate, and reduced levels of acetone, LDL, and VLDL [9]. Limited generalizability due to inclusion of patients only with hepatitis B cirrhosis is the major weakness of this study [9]. Elevated levels of creatine might be related to reduced metabolic and urinary clearance of creatine in patients with decompensated cirrhosis (e.g., presence of acute or chronic kidney disease) [9,13].

Using quantitative hepatic phosphorus-31 magnetic resonance spectrometry, Corbin et al. [12] reported that hepatic beta-ATP levels were significantly reduced in decompensated cirrhosis compared to compensated cirrhosis.

Collectively, results of these studies suggest alterations in the tricarboxylic acid cycle, dysfunction or deficiency of liver-specific phenylalanine hydroxylase that converts phenylalanine into tyrosine, altered amino acid metabolism and lipid synthesis in decompensated cirrhosis [9,12,14].

### 2.3. Differentiation between Severe Acute Alcoholic Hepatitis and Alcoholic Cirrhosis

Rachakonda et al. [15] conducted a prospective, case-control study of 25 patients with severe acute alcoholic hepatitis and 25 patients with alcoholic cirrhosis. Results of unbiased global serum metabolomic profiling showed a significant increase in the levels of 152 metabolites and decrease in the levels of 82 metabolites when patients with severe acute alcoholic hepatitis were compared with those with alcoholic cirrhosis [15]. These metabolites identified significantly dysregulated metabolic pathways including increased lipolysis, increased catabolism of branched chain amino acids by skeletal muscle, increased protein and peptide catabolism, decreased glucose utilization by glycolysis, altered fatty acid beta oxidation, intestinal dysbiosis, impaired glucuronidation and methylation when patients with severe acute alcoholic hepatitis were compared with those with alcoholic cirrhosis [15].

### 2.4. Differentiation between Cirrhosis Secondary to Alcoholic Hepatitis and Acute Decompensated Cirrhosis Secondary to Non-Alcohol Related Etiologies 

Ascha et al. [16] conducted a prospective study among 25 patients with cirrhosis secondary to alcoholic hepatitis and 23 patients with acute decompensated cirrhosis secondary to non-alcohol related etiologies. They found that betaine and citrulline, when used in combination, accurately differentiated patients with cirrhosis secondary to cirrhosis secondary to alcoholic hepatitis from the other group (area under the receiver operating characteristics curve = 0.835, 95% confidence interval: 0.747 to 0.978) [16].

### 2.5. Differentiation between Hepatitis B Cirrhosis and Alcoholic Cirrhosis

Using ^1^H NMR-spectroscopy, Qi et al. [10] differentiated 21 patients with hepatitis B cirrhosis from 20 patients with alcoholic cirrhosis (*R*^2^*Y* = 0.873, *Q*^2^*Y* = 0.826). Increased isobutyrate and creatine and decreased glutamate, glutamine and acetoacetate levels were observed in patients with hepatitis B compared with those with alcoholic cirrhosis [10].

### 2.6. Differentiation between Acute on Chronic Liver Failure and Chronic Liver Failure

A study conducted among 30 acute on chronic liver failure and 93 chronic liver failure (compensated or decompensated cirrhosis) patients using ^1^H NMR spectroscopy showed that an OPLS model was capable of differentiating patients with acute on chronic liver failure from those with chronic liver failure (*R*^2^*Y* = 0.63, *Q*^2^*Y* = 0.73) [17]. This model was correlated with a total of nine metabolites among which creatinine, glutamate, glutamine, ketone bodies including hydroxybutyrate and acetoacetate, pyruvate, lactate, phenylalanine and tyrosine showed an increased signal intensity, and HDL showed a decreased signal intensity [17]. Authors attributed the increased levels of ketone bodies and lactate to anaerobic glycolysis as the major energy source in hypoxia and hepatic necrosis, and the increased levels of glutamine and glutamate to dysfunction of hepatic urea metabolism [17].

### 2.7. Metabolomic Signature of Hepatorenal Dysfunction and Glomerular Filtration Rate in Patients with Cirrhosis

A unique metabolomic signature consisting of 34 metabolites that significantly increased in patients with high liver and kidney disease severity has recently been described in patients with cirrhosis [18]. Among these metabolites, 4-acetamidobutanoate, trans-aconitate, 1-methylhistidine, glucuronate, N4-acetylcytidine, 3-ureidopropionate, 3-methoxytyramine sulfate, cytidine, *S*-adenosylhomocysteine (SAH) and myo-inositol were the 10 most significantly increased ones when patients with high liver and kidney liver disease severity were compared with those with low liver and kidney disease severity [18]. This study that used ultrahigh performance liquid chromatography/tandem mass spectrometry to detect plasma metabolites, also showed that all signature metabolites were independent predictors of glomerular filtration rate measured by non-radiolabeled iothalamate plasma clearance in patients with cirrhosis; and erythronate had the highest significant association with measured glomerular filtration rate [18].

### 2.8. Metabolomic Profile in Patients with Cirrhosis and Minimal (Covert) Hepatic Encephalopathy

An ^1^H NMR-spectroscopy analysis conducted by Jimenez et al. [19] showed increased glucose, glycerol, lactate, methionine, trimethylamine-N-oxide and decreased acetoacetate, alanine, alpha-acid glycoproteins, branched chain amino acids, choline, glycine, and LDL levels in patients with cirrhosis and minimal hepatic encephalopathy (*n* = 39) compared with patients with cirrhosis without minimal hepatic encephalopathy (*n* = 62) (*R*^2^*Y* = 0.68, *Q*^2^*Y* = 0.63). A study conducted among 24 outpatients with cirrhosis and minimal hepatic encephalopathy by Saito et al. [20] identified the pre-treatment serum taurine as an independent predictor of response to treatment with l-carnitine (area under the receiver operating characteristics curve = 0.748, 95% confidence interval: 0.531 to 0.901).

### 2.9. Metabolomic Profile in Overt Hepatic Encephalopathy

A study conducted by Weiss et al. [21] among 14 patients with hepatic encephalopathy and 27 controls, identified several metabolites in the cerebrospinal fluid associated with altered brain energy metabolism pathways in patients with hepatic encephalopathy. The same study also identified plasma metabolites that differentiated patients with cirrhosis and hepatic encephalopathy from those without hepatic encephalopathy [21]. The key finding of this study was that metabolites associated with altered brain energy metabolism pathways that were detected in the cerebrospinal fluid of patients with hepatic encephalopathy were not detected in the plasma [21]. Small sample size and absence of cirrhosis in two patients with hepatic encephalopathy in the group whose CSF was examined were the major limitations of this study [21].

### 2.10. Impact of Lactobacillus GG and Rifaximin on Metabolome in Patients with Minimal Hepatic Encephalopathy

Bajaj et al. [22] conducted 8 weeks of a phase 1, randomized, placebo-controlled trial of lactobacillus GG administered twice daily. This study showed increased serum levels of hydroxylamine and benzoic acid and decreased serum levels of isoleucine, threonine and methionine and urine glycodeoxycholic acid, phophatidylcholines, vitamin C and riboflavin metabolites [22]. Despite these changes in the metabolome, there was no improvement in cognitive function [22].

In another study, Bajaj et al. [23] conducted an open label randomized clinical trial among 20 patients with cirrhosis and minimal hepatic encephalopathy to determine the mechanism of 8 weeks of rifaximin administered as 550 mg orally twice a day. Authors showed that rifaximin improved cognitive function and significantly changed serum metabolome, particularly with an increase in serum fatty acid levels, and a modest change in stool microbiome [23]. They also showed that patients who were treated with rifaximin had significantly increased levels of serum long chain fatty acids and reduced levels of lipopolysaccharides compared with controls [23]. They concluded that their findings were in line with those of previous studies that described the impact of fatty acids on brain function [24,25].

### 2.11. Metabolomic Profile in Hepatopulmonary Syndrome

Results of a prospective study of 24 patients with hepatopulmonary syndrome and 27 controls showed increased plasma levels of several primary and secondary bile acids, bilirubin, biliverdin, endothelin, fatty acids, nitric oxide synthase signaling regulators, sphingosine metabolites, urobilinogen and decreased plasma levels of monoglycerol [26].

### 2.12. Metabolomic Profile in Hepatocellular Carcinoma (HCC)

Several studies reported diagnostic and prognostic metabolites associated with HCC. Yang et al. [27], using ^1^H NMR-spectroscopy, showed that there were increased hepatic alanine, choline, glutamate, glutamine, glycine, lactate, leucine, and phosphorylethanolamine and decreased hepatic glucose, glycogen, and triglyceride levels in hepatic tissues involved with HCC compared to tissues free of HCC. Similarly, they showed increased hepatic alanine, choline, glutamate, glutamine, glycine, lactate, leucine, and phosphorylethanolamine and decreased glucose, glycerophophocholine, glycogen, phosphocholine, and triglycerides in high-grade HCC compared to low-grade HCC [27]. Gao et al. [11], using NMR-spectroscopy, showed significantly decreased levels of serum acetoacetate, choline, certain lipids (LDL, VDLD, unsaturated lipid), and valine along with increased levels of several metabolites including acetate, α-ketoglutarate, glycerol, 1-methylhistidine, glutamine, *n*-acetylglycoproteins, phenylalanine, pyruvate, and tyrosine in 39 patients with hepatocellular carcinoma compared to 63 healthy controls (*R*^2^*Y* = 0.515, *Q*^2^*Y* = 0.805). Collectively, these findings suggest an increase in glycolysis, alterations in tricarboxylic acid cycle, and impaired lipid synthesis [11,27].

Patterson et al. [28], using ultra performance liquid chromatography-electrospray ionization-quadrupole time-of-flight MS, showed decreased plasma lysophosphocholines (14:0; 20:3; and 22:6) and increased bilirubin and biliverdin levels in patients with cirrhosis and HCC compared with those without HCC. They also showed that plasma levels of several lysophosphocholines were decreased and levels of glycodeoxycholic acid and deoxycholic acid 3-sulfate were increased in patients with HCC compared with healthy controls [28]. In this study, the down-regulation of lysophosphocholines might be related to decompensation of liver disease rather than HCC per se, as similar findings were reported in patients with cirrhosis with increased mortality [29].

Lu et al. [30], using liquid and gas chromatography/MS, demonstrated that reduced acetylcarnitine levels accurately differentiated HCC-positive hepatic tissue from tumor-free hepatic tissues and predicted tumor grade and stage progression in HCC. Similarly, they found significantly reduced serum acetylcarnitine levels in patients with HCC compared to healthy subjects and those with HCC-free cirrhosis [30]. They suggested that acetylcarnitine can be considered a metabolomic biomarker complementary to alpha-fetoprotein levels in patients with HCC [30] and attributed reduced levels of acetylcarnitine in HCC patients to the reduced levels of carnitine observed in cancer cachexia [30,31,32].

Grammatikos et al. [33] reported that serum levels of long chain (C16–C20) and very long chain (≥C24) ceramides, sphingosine, sphinganine-1-phosphate and sphingosine-1-phosphate were significantly upregulated in 122 patients with cirrhosis and HCC when compared to 127 patients with cirrhosis without HCC. Results of this study suggest that ceramides play a major regulator role in hepatocarcinogenesis [33].

Soga et al. [34] performed metabolomic profiling of 248 serum samples by capillary electrophoresis/liquid chromatography/MS and reported that several γ-glutamyl dipeptides had large-fold increases in several liver diseases compared to healthy controls. The multivariate model that included γ-Glu-Ala, γ-Glu-Citrulline, γ-Glu-Thr, and γ-Glu-Phe accurately differentiated HCC from the cohort that included controls and patients with drug-induced liver injury, hepatitis B, hepatitis C and cirrhosis (for training cohort: Area under the receiver operating characteristics curve = 0.762, *p* = 0.00025; for validation cohort: Area under the receiver operating characteristics curve = 0.803) [34]. Authors attributed the increased levels of γ-glutamyl dipeptides to the increased production and consumption of reduced glutathione to overcome increased oxidative stress in hepatic injury [34].

### 2.13. Mortality without Liver Transplantation

Metabolomic analysis has also been used to predict mortality in patients with cirrhosis. In a study that included 103 patients with cirrhosis, the multivariate Cox regression analysis controlled for measured glomerular filtration rate and demographics showed that metabolites including *S*-adenosylhomocysteine, glucuronate, trans-aconitate, 3-ureidopropionate, 3-(4-hydroxyphenyl)lactate, 3-methoxytyramine sulfate, arabitol/xylitol, *N*-formylmethionine, phenyllactate and 7-methylguanine significantly predicted mortality in patients with cirrhosis [18].

McPhail et al. [29] identified several metabolites using ^1^H NMR spectroscopy and reversed-phase ultra-performance liquid chromatography coupled to time-of-flight MS in 80 patients with decompensated cirrhosis and validated them in an independent cohort of 101 patients with decompensated cirrhosis, 20 patients with stable cirrhosis and 47 healthy subjects. They reported that lysophophatidylcholines and phosphatidylcholines that play a key role in regulation of cell senescence, hepatic repair, immunomodulation and lipolysis were significantly decreased in non-survivors compared to survivors and were inversely correlated with cell death marker levels including M30 and M65 [29]. A prospective study that included 244 patients with cirrhosis showed that reduced levels of long and very long chain ceramides (e.g., C24-ceramide) were significantly associated with higher mortality without liver transplantation [35]. Similarly, another study showed several significantly reduced sphingomyelins, glycerophosphocholines and glycerophosphoethanolamines levels in patients with cirrhosis who had high Model for End-Stage Liver Disease-Sodium (MELD-Na) scores and diuretic-refractory ascites compared with those with less severe liver disease [18]. Collectively, the results of these studies suggest increased endoplasmic reticulum stress, membrane instability and impaired ceramide utilization and lipid signaling in worsening liver disease [18,36,37].

In a study conducted among 48 patients with cirrhosis, tyrosine itself and in combination with MELD score predicted 3-month and 6-month survival without liver transplantation with a greater accuracy compared with the MELD score alone [16].

## 3. Conclusions and Future Directions

Application of metabolomics in cirrhosis is broad and rapidly evolving. In this review, we presented several metabolomic studies conducted in patients with cirrhosis. Although these studies have identified multiple promising metabolites and/or metabolomic signatures to diagnose, differentiate and determine prognosis; only few of them interpreted data by performing pathway and network analyses to provide mechanistic and therapeutic insights in disease phenotypes. We recommend that following important steps should be considered when conducting metabolomics studies in patients with cirrhosis: (1) Clinical phenotypes related to etiology and complications of cirrhosis should be as distinctive as possible; (2) pathway and network analysis should be performed to identify the most altered pathways to discriminate among clinical phenotypes rather than simply providing a list of metabolites; (3) multi-omics, which is an integrative data analysis of metabolomics with proteomics, genomics, epigenomics, transcriptomics and microbiomics should be taken into consideration as it has recently gained ground to provide better mechanistic understanding of complex disease phenotypes in cirrhosis and its complications [38,39,40,41]; (4) metabolomic signatures should be validated in an independent cohort of patients with cirrhosis that has the same clinical phenotype as the training cohort. 

## Figures and Tables

**Table 1:** Major advantages and disadvantages of the mass spectrometry (MS) and NMR Spectroscopy.

Mass Spectrometry	NMR Spectroscopy
More sensitivity	Less sensitivity
Requires a smaller amount of sample	Requires more samples
Destructive to the sample	Non-destructive to the sample
Various ionization techniques applied to detect a greater number of metabolites	Single method applied
All elemental composition	Proton, carbon, phosphorus
Less expensive	More expensive
Difficult to measure polymers	Great advantage for polymer analysis
Less reproducible	More reproducible
Equipment requires smaller space	Equipment requires larger space

**Table 2:** Current applications of metabolomics in patients with cirrhosis.

Applications	Representative Metabolites ^*^		Representative Studies	Technique Used
	Increased ^**^	Decreased ^**^		
Differentiation Between Patients with Cirrhosis ** and Healthy Controls	Blood acetate, α-ketoglutarate, glycerol, glutamine, 1-methylhistidine, *N*-acetylglycoproteins, phenylalanine, pyruvate, taurine, tyrosine, hepatic phosphomonoester/phosphodiester ratio	Blood acetoacetate, choline, isoleucine, LDL, leucine, unsaturated lipid, valine, VLDL, hepatic tissue beta-ATP	Gao et al. (2009) [11], Corbin et al. (2004) [12]	^1^H NMR-spectroscopy, quantitative hepatic phosphorus-31 magnetic resonance spectroscopy
Differentiation Between Decompensated Cirrhosis ** and Compensated Cirrhosis	Blood alanine, creatine, glutamate, glutamine, histidine, lysine, phenylalanine, pyruvate, succinate	Blood acetone, LDL, VLDL, hepatic tissue beta-ATP	Qi et al. (2012) [9], Corbin et al. (2004) [12]	^1^H NMR-spectroscopy, quantitative hepatic phosphorus-31 magnetic resonance spectroscopy
Differentiation Between Severe Acute Alcoholic Hepatitis ** and Alcoholic Cirrhosis	Blood glucuronate, biliverdin, erythronate, methionine, lactate, cortisol, *N*-acetyltryptophan, symmetric dimethylarginine	Blood choline, glycerophosphocholine (GPC), glycerol-3-phosphate, ascorbate, serotonin, isoleucine, leucine, valine, deoxycholate, glycodeoxycholate	Rachakonda et al. (2014) [15]	MS
Differentiation Between Hepatitis B Cirrhosis ** and Alcoholic Cirrhosis	Blood creatine, isobutyrate	Blood acetoacetate, glutamate, glutamine	Qi et al. (2012) [10]	^1^H NMR-spectroscopy
Differentiation Between Cirrhosis Secondary to Alcoholic Hepatitis ** and Acute Decompensated Cirrhosis Secondary to Non-Alcohol Related Etiologies	Blood betaine, citrulline, creatinine, phenylalanine, homocitrulline, tyrosine, octenoyl-carnitine, symmetric dimethylarginine		Ascha et al. (2016) [16]	MS
Differentiation Between Acute on Chronic Liver Failure ** and Chronic Liver Failure	Blood creatinine, glutamate, glutamine, ketone bodies (hydroxybutyrate and acetoacetate), pyruvate, lactate, phenylalanine and tyrosine	Blood HDL	Amathieu et al. (2014) [17]	^1^H NMR-spectroscopy
Metabolomic Signature of Hepatorenal Dysfunction in Cirrhosis (Differentiation Between High Liver and Kidney Disease Severity ** and Low Liver and Kidney Disease Severity)	The top 10 among 34 blood metabolites based on fold increase included 4-acetamidobutanoate, trans-aconitate, 1-methylhistidine, glucuronate, N4-acetylcytidine, 3-ureidopropionate, 3-methoxytyramine sulfate, cytidine, *S*-adenosylhomocysteine, myo-inositol		Mindikoglu et al. (2017) [18]	MS
Metabolomic Signature of Reduced Glomerular Filtration Rate	The top 10 among 34 blood metabolites based on R-square included erythronate, N6-carbamoylthreonyladenosine, 1-methylhistidine, pseudouridine, *N*-acetylserine, creatinine, 7-methylguanine, N2–N2-dimethylguanosine, C-glycosyltryptophan, myo-inositol		Mindikoglu et al. (2018) [18]	MS
Minimal (Covert) Hepatic Encephalopathy	Blood glucose, glycerol, lactate, methionine, trimethylamine-N-oxide	Acetoacetate, alanine, alpha-acid glycoproteins, branched chain amino acids, choline, glycine, and lipid moiety, taurine	Jimenez et al. (2010) [19], Saito et al. (2016) [20]	^1^H NMR-spectroscopy, MS
Differentiation Between Subjects with Hepatic Encephalopathy ** and Controls without Neurological Disease	Cerebrospinal fluid glutamine, glutamate, phenylalanine, tryptophan, methionine, formyl-methionine, N4-acetylcytidine	Cerebrospinal fluid taurine	Weiss et al. [21]	MS
Impact of Lactobacillus GG on Metabolome in Patients with Minimal Hepatic Encephalopathy	Blood hydroxylamine and benzoic acid	Blood isoleucine, threonine, methionine, urine metabolites including glycodeoxycholic acid, phophatidylcholines, vitamin C, riboflavin metabolites	Bajaj et al. (2014) [22]	MS
Impact of Rifaximin on Metabolome in Patients with Minimal Hepatic Encephalopathy	Blood myristic acid, caprylic acid, palmitic acid, succinic acid, fructose	Blood lipopolysaccharides	Bajaj et al. (2013) [23]	MS
Hepatopulmonary Syndrome	Blood primary and secondary bile acids, bilirubin, biliverdin, endothelin, fatty acids, nitric oxide synthase signaling regulators, sphingosine metabolites, urobilinogen	Blood monoglycerol	Fallon et al. (2015) [26]	MS
Differentiation Between Hepatocellular Carcinoma (HCC) ** and HCC-Free Hepatic Tissue	Hepatic alanine, choline, glutamate, glutamine, glycine, lactate, leucine, and phosphorylethanolamine	Hepatic glucose, glycogen, and triglyceride, acetylcarnitine	Yang et al. (2007) [27], Lu et al. (2016)	^1^H NMR-spectroscopy, MS
Differentiation Between High-Grade HCC ** and Low-Grade HCC	Hepatic alanine, choline, glutamate, glutamine, glycine, lactate, leucine, and phosphorylethanolamine	Hepatic glucose, glycerophophocholine, glycogen, phosphocholine, and triglycerides	Yang et al. (2007) [27]	^1^H NMR-spectroscopy
Differentiation Between Cirrhosis with HCC ** and Cirrhosis without HCC	Blood bilirubin, biliverdin, γ-glutamyl dipeptides (γ-Glu-Ala, γ-Glu-Citrulline, γ-Glu-Thr, and γ-Glu-Phe), long chain (C16–C20) and very long chain (≥C24) ceramides, sphingosine, sphinganine-1-phosphate and sphingosine-1-phosphate	Several blood lysophosphocholines	Patterson et al. (2011) [28], Soga et al. (2011) [34], Grammatikos et al. (2016) [33]	MS
Differentiation Between Subjects with HCC ** and Healthy Subjects	Blood glycodeoxycholic acid, deoxycholic acid 3-sulfate, acetate, α-ketoglutarate, glycerol, 1-methylhistidine, n-acetylglycoproteins, phenylalanine, pyruvate, tyrosine	Several blood lysophosphocholines, acetoacetate, choline, certain lipids (LDL, VLDL, unsaturated lipid), and valine	Patterson et al. (2011) [28], Gao et al. (2009) [11]	^1^H NMR-spectroscopy, MS
Increased Mortality without Liver Transplantation	Blood *S*-adenosylhomocysteine, glucuronate, trans-aconitate, 3-ureidopropionate, 3-(4-hydroxyphenyl)lactate, 3-methoxytyramine sulfate, arabitol/xylitol, *N*-formylmethionine, phenyllactate and 7-methylguanine, tyrosine	Several blood sphingomyelins, glycerophosphocholines, glycerophosphoethanolamines, lysophophatidylcholines, phosphatidylcholines, long and very long chain ceramides (e.g., C24-ceramide)	Mindikoglu et al. (2018) [18], McPhail et al. (2016) [29], Grammatikos et al. (2015) [35], Ascha et al. (2016) [16]	MS, ^1^H NMR-spectroscopy

* Metabolites included in this table do not contain the full list of metabolites reported in the representative studies cited. ** Increased and decreased levels of metabolites occurred in the condition indicated.
